# Supplementary material for: Phosphate availability and implications for life on ocean worlds
Source: Nat Commun. 2023 Apr 25;14:2388. doi: 10.1038/s41467-023-37770-9 (PMC10130162; doi:10.1038/s41467-023-37770-9)
Supplement: Supplementary file 1 — Supplemental Information [file 41467_2023_37770_MOESM1_ESM.pdf]

Supplemental information for "Phosphate availability and implications for life on ocean worlds"

Noah Randolph-Flagg[1,2,3], Tucker Ely[2,4,5], Sanjoy M. Som[1,3], Everett L. Shock[5], Christopher R. German[6], Tori M. Hoehler[1]

[1] Space Science and Astrobiology Division, NASA Ames Research Center, Moffett Field, CA, USA.

[2] NASA Postdoctoral Program, Universities Space Research Association, Columbia, MD, USA.

[3]Blue Marble Space Institute of Science, Seattle, WA, USA.

[4] Department of Earth and Environmental Sciences, University of Minnesota, Minneapolis, MN, USA.

[5] School of Earth and Space Exploration, Arizona State University, Tempe, AZ, USA.

[6] Marine Chemistry and Geochemistry, Woods Hole Oceanographic Institution Woods Hole, MA 02540, USA

Corresponding author: Noah Randolph-Flagg, nrflagg@berkeley.edu

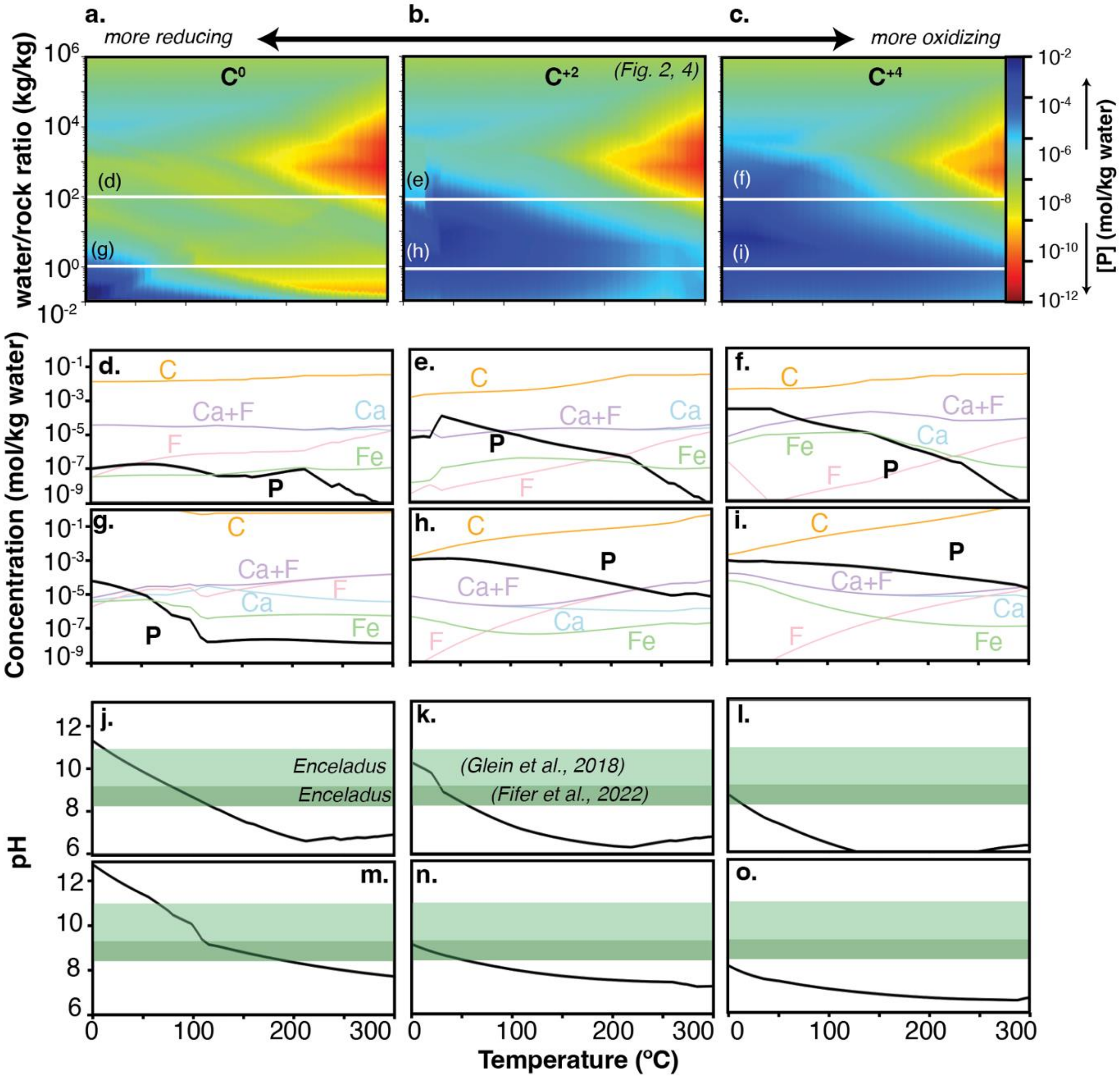

Supplemental Figure 1. Additional fluid chemistry from Fig. 3 highlighting relationship between P, Ca, and pH. Comparison of [P] (**a-c**), dissolved aqueous species (**d-i**), and pH (**j-o**) under the three conditions considered in the redox sensitivity analysis (main text Fig. 2). Panels **d, e, f, j, k, l** and **g, h, i, m, n, o** respectively correspond to "slices" taken at W:R = 100 and W:R = 1. Inferred Enceladus ocean pH range of 8-9 from Fifer and colleagues[1] is highlighted in dark green and 8-11 from Glein and colleagues[2] in light green.

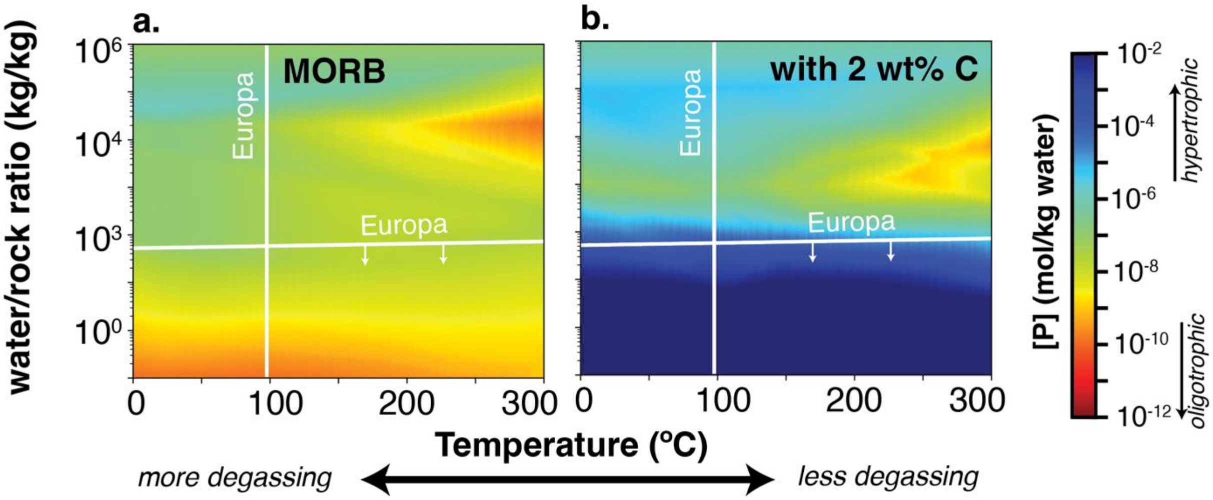

Supplemental Figure 2. Effect of silicate melting and degassing on dissolved phosphate concentrations. On large planetary bodies (such as Earth or Europa) crustal differentiation can reallocate the distribution of elements within the rocky interior. In general, this take two forms– 1) the segregation of siderophile elements (Fe, Ni, S) into a metallic core and 2) the production of volcanic fluids (primarily basalts and hydrothermal fluids such as CO_2 and H_2S.) Reacting the most common rock on Earth's surface, mid-ocean ridge basalt or MORB, (a) with the minimal fluid used in chondrite simulations (Figs. 2 and 3) produces phosphate concentrations significantly lower than with CI chondrite reactant, but still within a range ($>10^{-9}$ mol/kg) that can be utilized by phosphate-limited microorganisms on Earth. Including the carbon liberated by crustal melting of CI chondrite (2 wt%)[3] in the otherwise identical MORB reaction model (b) produces very high phosphate concentrations relative to Earth environments ($>10^{-4}$ mol/kg water). This sensitivity highlights the importance of considering chemical evolution at a system-level, rather than in a MORB-only calculation, and suggests a key direction for future research on P availability on larger bodies, where magmatic processing may occur.

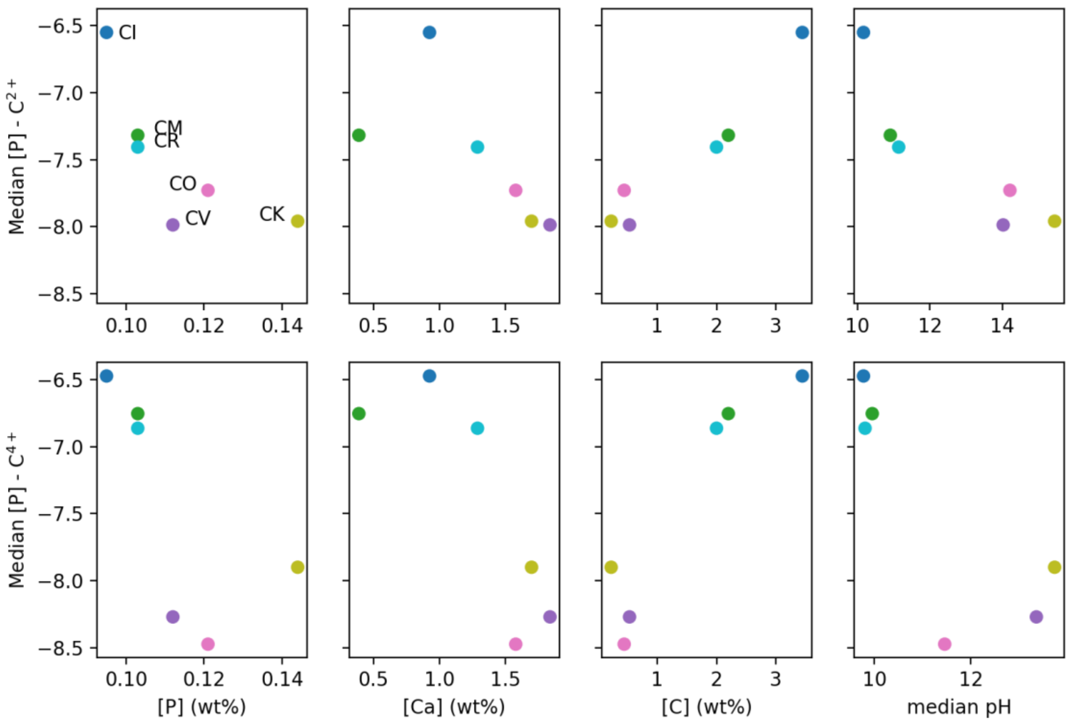

Supplemental Figure 3. Comparison of median phosphate concentrations for all temperature and water/rock ratios for each chondrite type (a total of ~1.9 million aqueous chemistry simulations) as a function of input parameters (Supp. Table 1) for each chondrite type. Fig. 2 shows the full water/rock ratio temperature space of each chondrite type.

Supplemental Table 1. Average weight percentage for elemental abundance in chondrites (n = 1800, [P] = 0.11 ± 0.02 wt%[3]) and mid ocean ridge basalts (n = 3598, [P] = 0.074 ± 0.04 wt%[4].) Note that the MORB values listed are renormalized to the stoichiometry used for this study which leads to small change relative Fig. 1a.

	CI	CM	CV	CO	CK	CR	MORB	CI^0	CI^{+4}
**Si**	10.64	12.7	15.7	15.8	15.8	15	24.1	10.64	10.64
**Al**	0.865	1.13	1.68	1.4	1.47	1.15	7.95	0.865	0.865
**Fe**	18.2	21.3	23.5	25	23	23.8	8.29	18.2	18.2
**Mg**	9.7	11.5	14.3	14.5	14.7	13.7	4.65	9.7	9.7
**Ca**	0.926	1.29	1.84	1.58	1.7	1.29	8.31	0.926	0.926
**Na**	0.5	0.39	0.34	0.42	0.31	0.33	2.12	0.5	0.5
**K**	0.055	0.037	0.036	0.036	0.029	0.0315	0.135	0.055	0.055
**S**	5.41	2.7	2.2	2.2	1.7	1.9	0.106	5.41	5.41
**Cl**	0.5	0.39	0.34	0.42	0.31	0.33	0	0.5	0.5
**P**	0.095	0.103	0.112	0.121	0.11	0.103	0.082	0.095	0.095
**Mn**	0.194	0.165	0.152	0.162	0.144	0.166	0	0.194	0.194
**Li**	0.00015	0.00015	0.00017	0.00018	0.00014	0	0	0.00015	0.00015
**F**	0.006	0.0038	0.0024	0.003	0.002	0	0	0.006	0.006
**C**	3.45	2.2	0.53	0.44	0.22	2	0.028	3.45	3.45

Supplemental Table 2. Thermodynamic data from Ely and references therein[6] used for equilibrium EQ3/6[5,6] models. Sensitivity analyses also were performed for a standard software standard (cmp), Yucca Mountain (ymp), and our curated database modified for iron phosphates (cst).

a. Aqueous species

AlH_2PO_4	$FePO_4$	HPO_4	$MnH2PO_4$	PO_3F
$CaPO_4$	$H2PO_3F$	$KHPO_4$	$MnHPO_4$	PO_4
$FeH2PO_4$	$H2PO_4$	$MgH2PO_4$	$MnPO_4$	
$FeHPO_4$	$H3PO_4$	$MgHPO_4$	$NaHPO_4$	
$FeHPO_4$	HPO_3F	$MgPO_4$	$PbHPO_4$	

b. Mineral species

**Mineral name**	**Mineral formula**	**Earth abundance**	**Chondrite**	**Database**
hydroxylapatite	$Ca_5(PO_4)3OH$	abundant	x	all
fluoroapatite	$Ca_5(PO_4)3F$	abundant	x	all
strengite	$(Fe^{+3})(PO_4)*2H_2O$	somewhat		cst, cmp, ymp
vivianite	$(Fe^{+2})_3(PO_4)_2*8H_2O$	somewhat	x	cst, cmp, ymp
heterocite	$(Fe^{+3})PO_4$	rare		cst, cmp, ymp
whitlockite	$Ca_3(PO_4)_2$	somewhat		cst, cmp, ymp
berlinite	$AlPO_4$	rare		cst, cmp, ymp
brushite	CaHPO4:2H2O	rare		ymp
corkite	$PbFe_3(PO_4)(SO_4)(OH)_6$	rare		cmp
Ni phosphate	$Ni_3(PO_4)_2$	rare		cmp
				cmp, ymp
U, Pb, Pu phosphate	$X(PO_4)_2$	rare		

c. Thermodynamic data

LogK @ Psat	0	25	60	100	150	200	250	300
**hydroxylapatite**	-0.2732	-3.0746	-6.9552	-11.1769	-16.2217	-21.2242	-26.4821	-32.5216
**fluoroapatite**	-23.8547	-24.994	-27.065	-29.7301	-33.3622	-37.3831	-42.0032	-47.7388
**strengite**		-11.3429						
**vivianite**		-4.7237						
**heterocite**		-7.3611						
**whitlockite**		-4.2249						

Supplemental References

1. Fifer, L., Catling, D., & Toner, J., Chemical fractionation modeling of plumes indicates a gas-rich, moderately alkaline Enceladus ocean. The Planetary Science Journal, **3** (8), 191 (2022).
2. Glein, C., Postberg, F., & Vance, S., The geochemistry of Enceladus: composition and controls. Enceladus and the icy moons of Saturn, **39**, (2018).
3. Hezel, D., MetBase.org as a Research and Learning Tool for Cosmochemistry. Elements: An International Magazine of Mineralogy, Geochemistry, and Petrology, **16** (1), 73-75, (2020).
4. Ely, T., Thermodynamic Cartography in Basalt-Hosted Hydrothermal Systems, Dissertation, Arizona State University, (2020).
5. Wolery, T., & Jarek, R., Software user's manual. EQ3/6, version, **8**, 376, (2003).
6. Wolery, T., & Jove-Colon, C., Qualification of thermodynamic data for geochemical modeling of mineral-water interactions in dilute systems (No. ANL-WIS-GS-000003 REV 00). YMP (Yucca Mountain Project, Las Vegas, Nevada), (2004).
